# Persistent compression of uterine insertion on maternal blood dynamic change in fetoscopic laser photocoagulation surgery: a novel method

**DOI:** 10.1186/s12884-026-09227-6

**Published:** 2026-05-13

**Authors:** Huirong Tang, Xingbo Tian, Chenyan Dai, Ya Wang, Yuan Wang, Liang Jin, Xian Xiao, Gongli Chen, Mingming Zheng

**Affiliations:** 1 Department of Obstetrics and Gynecology, Affiliated Hospital of Medical School, Nanjing Drum Tower Hospital, Nanjing University, Nanjing, 210008 China; 2 The Third Obstetrics Department (Fetal Medicine Center), Chongqing Health Center for Women and Children, Women and Children’s Hospital of Chongqing Medical University, No. 64 Jintang Street, Qixinggang, Yuzhong District, Chongqing, China; 3 Fetal Medicine center, Anhui Women and Children’s Medical Center, Yimin Street No.15, Hefei, Anhui China

**Keywords:** Blood dynamic change, Fetoscopic laser photocoagulation, Compression

## Abstract

**Background:**

Fetoscopic laser photocoagulation (FLP) has been performed as first-line approach for twin-to-twin transfusion syndrome (TTTS) in monochorionic twin pregnancies. The majority of researches are focusing on advances in fetal treatment and long-term outcome, few researches on maternal surgery-related complications are reported. This study is to compare the maternal blood dynamic changes in women with and without persistent compression on the uterine insertion after the FLP procedure.

**Methods:**

This was a retrospective study conducted at two tertiary referral centers in China between November 2018 and July 2023. TTTS-cases undergoing FLP surgery were enrolled and divided into two groups upon whether having compression on the uterine insertion after FLP. The changes of maternal hemoglobin, hematocrit before and after the procedure were compared between the two groups.

**Results:**

A total of 111 TTTS-cases were finally analyzed including 46 cases with persistent compression and 65 cases without persistent compression. The two groups had similar values of hemoglobin (10.7 ± 1.1 versus 10.5 ± 1.2 g/dl, *p* = 0.513) and hematocrit (32.3 ± 3 versus 31.3 ± 3.2, *p* = 0.099) before FLP. A similar volume of amniodrainage during the surgery was observed in the two groups (710 ml versus 850 ml, *p* = 0.255). The decrease in hemoglobin in TTTS-cases with persistent compression was significantly lower than that in TTTS cases without continuous compression (1.1 g/dL versus 1.5 g/dL, *p* = 0.014). A decrease trend in change of hematocrit was observed between cases with persistent compression (3.1%) and those without persistent compression (4.0%), while it did not achieve a significant difference (*p* = 0.050). The blood transfusion rate in TTTS-cases with and without compression was 2.2% and 7.7% respectively (*p* = 0.205).

**Conclusion:**

The decrease in hemoglobin and hematocrit could be mitigated by persistent compression on the uterine insertion site after FLP, which is an economical, simple and convenient method.

**Supplementary Information:**

The online version contains supplementary material available at 10.1186/s12884-026-09227-6.

## Background

Over the past 25 years, fetoscopic laser photocoagulation (FLP) has been performed as first-line approach for twin-to-twin transfusion syndrome (TTTS) in monochorionic twin pregnancies, and many studies have shown that FLP can improve the neonatal outcome compared with other conventional treatments [1–2]. The majority of researches are focusing on fetal short- and long-term outcome post-laser surgery [3–5], but less is known about maternal complication. De Lia et al. were the first to report maternal hemodilution in pregnancies complicated by TTTS after laser surgery and amniodrainage, suggesting that such procedure could have an impact on maternal circulation [6]. The pathophysiology of maternal hemodilution have not yet been clarified, but several studies have observed that intrauterine interventions can lead to decompression of the uterine and abdominal compartments, subsequently contributing to hemodilution [7–9].

Another notable maternal complications associated with FLP is bleeding from the uterine insertion site into the abdominal cavity. According to Sacco A’s review, bleeding during the procedure was noted in 1.74% of fetoscopic surgery cases (95% CI, 1.25-2.32) [10]. As high as 10% of uterine wall bleeding rate was reported after FLP, especially in cases of general anaesthesia [11]. This complication is usually treated conservatively without invasive management. While, in some severe cases, blood transfusion and even hysterectomy are required [12, 13]. Intraoperative blood transfusion was required in 0.27% undergoing fetoscopic surgery (95% CI, 0.18‐0.38) [10].

To mitigate significant maternal hemodilution resulting from rapid intrauterine decompression and to reduce potential bleeding from uterine puncture sites, we developed a novel method involving immediate, firm compression at the endoscopic insertion site using gauze and bandage after FLP. The proposed mechanism of this technique is twofold. First, the external persistent compression helps to stabilize intra-abdominal and uterine pressure, reducing the sudden hemodynamic changes that follow rapid decompression. Second, direct pressure on the puncture tract through the abdominal wall and uterus achieves hemostasis by slowing bleeding and promoting clot formation.

In this study, we compared changes in maternal hemoglobin and hematocrit levels between women who received persistent compression and those who did not, aiming to evaluate whether this intervention reduces hemodynamic alterations following FLP surgery.

## Methods

### Study design and participants

This was a retrospective study conducted at two tertiary referral centers in China (Nanjing Drum Tower Hospital (DTH) and Chongqing Health Center for Women and Children (CQH)) between November 2018 and July 2023. All consecutive monochorionic, diamniotic twin pregnancies up to 26 weeks’ gestation complicated by TTTS (Quintero stage 2, 3, or 4) and women with Quintero stage 1 with clinical symptoms due to polyhydramnios treated with FLP were included. Cases were excluded when blood test results before or one day after surgery and the information on delivery were not available. The study was approved by the Ethics Committee of Nanjing Drum Tower Hospital and Chongqing Health Center for Women and Children (2013058, 2020YL2002) and each center’s respective institutional review board. The study was carried out based on the ethical standards of the 1964 Declaration of Helsinki and its later amendments.

### Surgical procedure

**Fig. 1:**
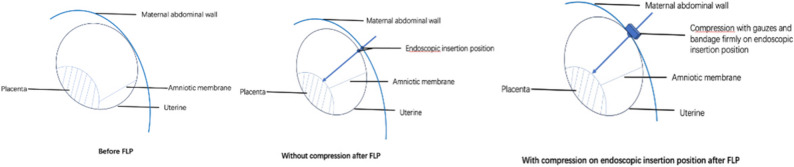
The schematic diagram of the compression of the uterine

**Fig. 2:**
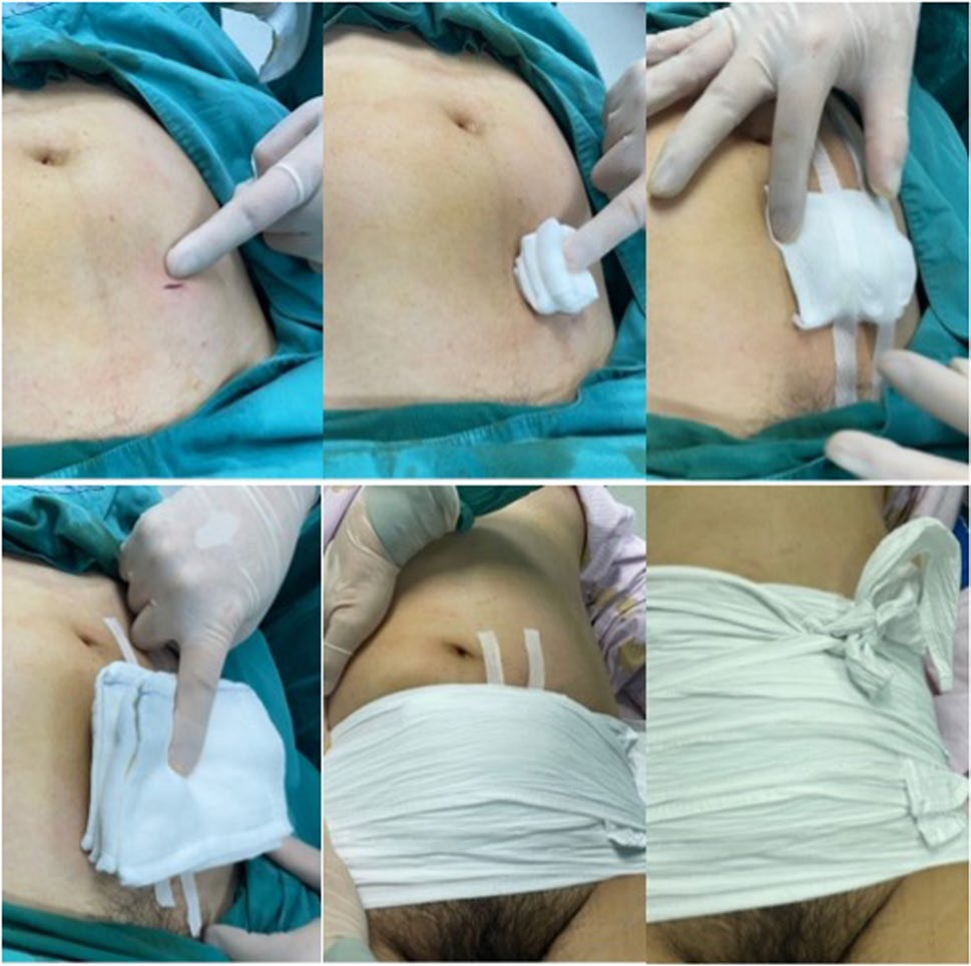
The procedure of the compression after fetoscopic laser photocoagulation surgery

Patients routinely received indomethacin (12.5 mg) transrectally. Prophylactic intravenous antibiotics cefazolin (2.0 g) with 100 ml 0.9% sodium chloride or clindamycin (0.9 g) with 250 ml 0.9% sodium chloride for women with cefazolin allergy half an hour before the procedure. IAll fetoscopic laser procedures were undertaken by experienced operators (Mingming Zheng and Gongli Chen). A cannula was introduced transabdominally into the amniotic cavity of the recipient twin by sharp trocar insertion under ultrasound visualization to avoid puncturing the large blood vessels of the abdominal wall after local anaesthesia by lidocaine. FLP was performed using a 3.3 mm fetoscope (Karl Storz, Tuttlingen, Germany) with a cannula of 10 French and a 600 μm laser fiber connected to a diode or Nd: YAG laser device (Dornier MedTech, Wessling, Germany). The laser procedure was followed by draining the excessive amniotic fluid to the deepest pocket of 6–8 cm. Immediately after removing the fetoscopy, the entry point including maternal skin and uterine wall was assessed to exclude active hemorrhage by ultrasound. Oral tocolysis with 10 mg nifedipine was administered three times daily for 48 h in the case of clinically apparent uterine contractions after the surgery. TTTS-cases in CQH were routinely treated without persistent compression after FLP as well as cases in DTH between November 2018 and July 2021. Since August 2021 in DTH, persistent compression hemostasia was used immediately after removal of the laser with gauzes and bandage on uterine insertion for about 6 h after FLP. TTTS-cases were divided into two groups upon whether having compression after FLP. The Figs. 1 and 2 showed the details of the compression.

### Outcome parameters

We collected the following variables from ultrasound records and medical records: maternal characteristics, TTTS stage, placental location, gestational age at laser (in weeks), laser techniques, volume of amniotic fluid reduction. Maternal serum hemoglobin (Hb) and hematocrit (Hct) were measured at admission (usually 6–24 h before the intervention) and 24 h after the intervention. The primary outcome was the change of hemoglobin and hematocrit of maternal blood. The secondary outcome was maternal blood transfusion during and after FLP.

### Statistics

Data were tested for normal distribution using the Kolmogorov–Smirnov and Shapiro–Wilk tests. Correlation analyses between related parameters were performed with Spearman ´s rho if a dataset was not normally distributed. Not-related parameters were tested with the Wilcoxon test. In the case of normal distribution, Student’s t-test was performed. Maternal hemoglobin and hematocrit before and after fetoscopy were performed by paired t-test. A probability of *p* < 0.05 was considered statistically significant. Analyses were carried out using SPSS Statistics 26.0 (IBM, Armonk, NY, USA).

## Results

**Fig. 3:**
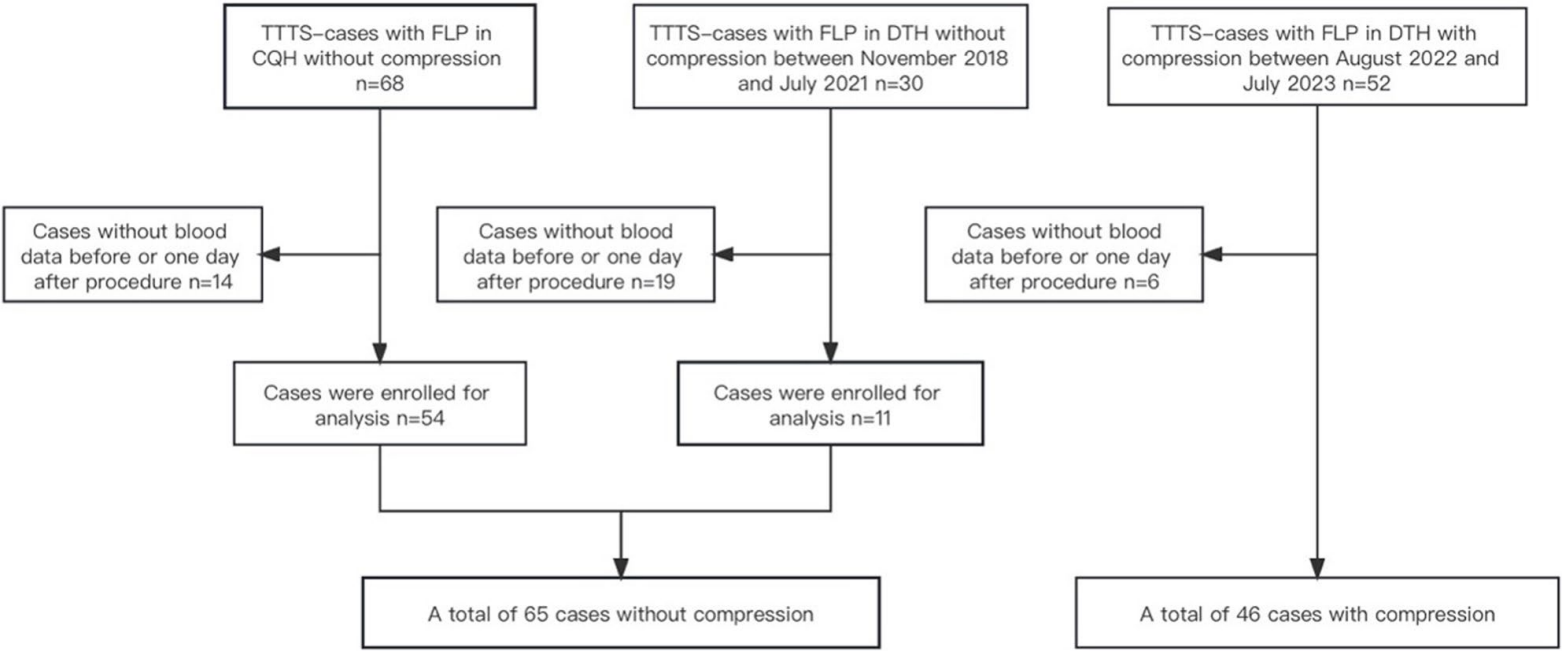
Flowchart of the study

A total of 68 cases with TTTS in CQH and 82 cases in DTH undergoing FLP were recruited. Thirty-nine of 150 (26.0%) cases were excluded because of unavailable maternal blood results. Thus, 54 (79.4%) cases in CQH and 57 (69.5%) cases in DTH were finally enrolled for analysis. They were further divided into two groups, Group 1 of 46 cases with persistent compression and Group 2 of 65 cases without persistent compression (Fig. 3).

### Comparison of characteristics of TTTS-cases with and without persistent compression on uterine insertion after FLP

The characteristics of women treated with FLP in the two groups were displayed in Table 1. We found no significant difference between the two groups with respect to maternal age (29.0 versus 30.0 years, *p* = 0.768), gestational age at laser (22.0 ± 2.9 versus 21.9 ± 2.9 weeks, *p* = 0.848), proportion of TTTS stages (*p* = 0.057) and the proportion of placenta anterior position (50.0% versus 56.3%, *p* = 0.237). There was also no significant difference between the volume of amniodrainage. The median volume of amniodrainage was 710 mL (100–2700mL) and 850 mL (150-3000mL) at the surgery respectively. No significant difference was observed in aspect of the delivery gestational weeks between the two groups.

**Table 1:** The characteristics of TTTS-cases with and without compression on uterine insertion after FLP

Parameters	With compression (Group 1) *N* = 46	Without compression (Group 2) *N* = 65	*P* value
Maternal age, years, Median, range	29.0 (23.0–42.0)	30.0 (22.0–39.0)	0.768
Gestational weeks at procedure Mean, SD	22.0, 2.9	21.9, 2.9	0.848
Indications of the surgery			0.057
TTTS stage 1 n, %	11/46, 23.9	4/65, 6.2	
TTTS stage 2 n, %	16/46, 34.8	31/65, 47.7	
TTTS stage 3 n, %	8/46, 17.4	12/65, 18.5	
TTTS stage 4 n, %	11/46, 23.9	18/65, 27.7	
Placenta position			0.237
Anterior n, %	19/38, 50.0	36/64, 56.3	
Posterior n, %	19/38, 50.0	28/64, 43.8	
Volume of AF reduction, mL Median, range	710 (100–2700)	850 (150–3000)	0.255
GW at delivery, weeks, Mean, SD	32.1,5.1	33.2,3.2	0.277

*TTTS* Twin-to-twin transfusion syndrome, *FLP* Fetoscopic laser photocoagulation, *AF* Amniotic fluid, *GW* Gestational week, *SD* Standard deviation

### Comparison of maternal hemodynamic change in TTTS-cases with and without persistent compression on endoscopic insertion after FLP

Before FLP, the two groups showed comparable values of hemoglobin (10.7 ± 1.1 versus 10.5 ± 1.2 g/dl, *p* = 0.513) and hematocrit (32.3 ± 3 versus 31.3 ± 3.2, *p* = 0.099). After FLP, significant differences were observed in hemoglobin (9.6 ± 1.1 versus 9.1 ± 1.2 g/dl, *p* = 0.025) and hematocrit (29.3 ± 3.1 versus 27.3 ± 3.2, *p* = 0.002) between TTTS-cases with and without persistent compression respectively. What’s more, the decrease in hemoglobin in TTTS-cases with persistent compression was significantly lower than that in TTTS-cases without persistent compression (1.1 g/dL versus 1.5 g/dL, *p* = 0.014). Although similar trends were observed for reductions in hematocrit (3.1% vs. 4.0%, *p* = 0.050) and the need for blood transfusion (2.2% vs. 7.7%, *p* = 0.205), these did not reach statistical significance (Table 2).

**Table 2:** The values of hemoglobin and hematocrit before and after FLP in women with and without compression

Parameters	With compression	Without compression	*P* value
Hemoglobin before FLP, g/dl, mean, SD	10.7, 1.0	10.6, 1.2	0.513
Hemoglobin after FLP, g/dl, mean, SD	9.6, 1.1	9.1,1.2	0.025
Change of Hemoglobin, g/dl, mean, SD	1.1, 0.8	1.5, 0.7	0.014
Hematocrit before FLP, %, mean, SD	32.3, 3.0	31.3, 3.2	0.099
Hematocrit after FLP, %, mean, SD	29.3, 3.1	27.3, 3.2	0.002
Change of Hematocrit, %, mean, SD	3.1, 2.5	4.0, 2.5	0.050
Blood Transfusion after the procedure n, %	1/46, 2.2	5/65, 7.7	0.205

*FLP* Fetoscopic laser photocoagulation, *SD* Standard deviation

To minimize potential inter-hospital and inter-operator variability, a subgroup analysis was conducted within Drum Tower Hospital, including 46 compression cases and 11 non-compression cases. As shown in Supplementary Table 1, the decrease in hemoglobin remained significantly smaller in the persistent compression group (1.1 g/dL vs. 1.8 g/dL, *p* = 0.019), while the reduction in hematocrit demonstrated a similar trend without reaching statistical significance (3.1% vs. 4.9%, *p* = 0.054).

Additionally, to address the potential influence of operator learning-curve effects, we performed an inter-center comparison restricted to non-compression cases. The declines in hemoglobin and hematocrit were comparable between DTH (before August 2021) and CQH (Supplementary Table 2), indicating that the early-stage DTH procedures had already reached a stable performance level. This supports that the improved hemodynamic outcomes observed in the compression group were unlikely to be explained by surgical experience alone.

### The correlation between the volume of amniodrainage at surgery and changes of maternal serum hemoglobin

**Fig. 4:**
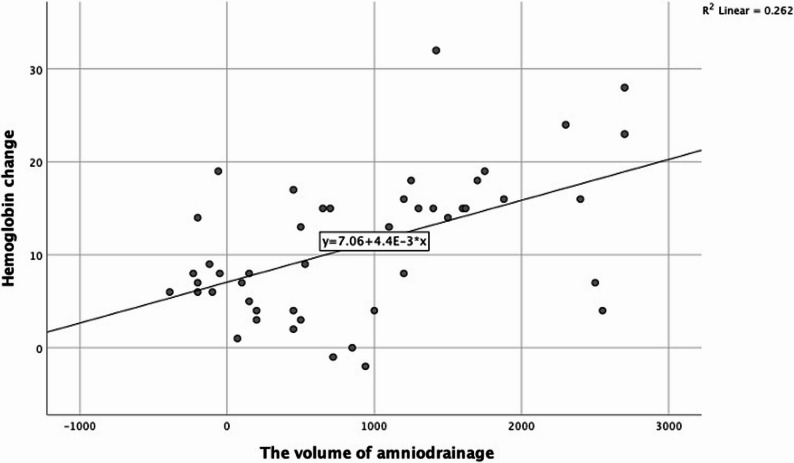
Correlation between the volume of amniodrainage at surgery and changes of maternal serum hemoglobin

**Fig. 5:**
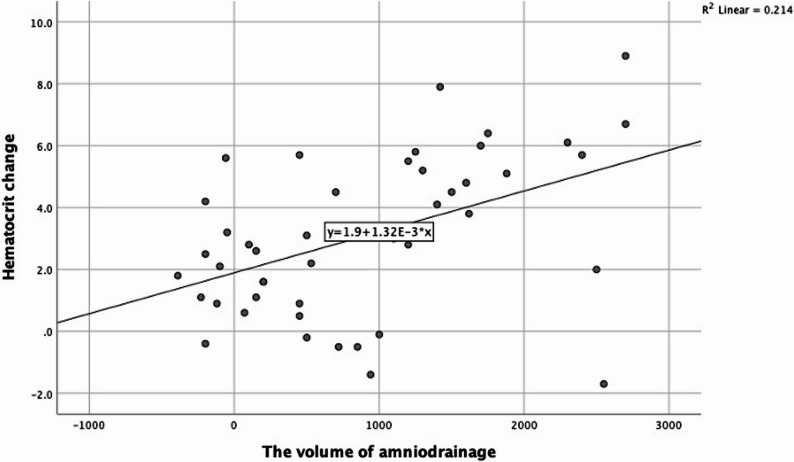
Correlation between the volume of amniodrainage at surgery and changes of maternal serum hematocrit

There was a significant correlation between the volume of amniodrainage and the effects on maternal blood characteristics. Within a 24 h interval, there was a positive correlation between hemoglobin (Spearman’s rho 0.262; *p* = 0.004) (see Fig. 4), Hematocrit (Spearman’s rho 0.214; *p* < 0.001) (see Fig. 5) and the amount of amniodrainage during the intervention.

## Discussion

The decline in maternal hemoglobin and hematocrit following FLP is generally attributed to postoperative hemodilution. Previous studies have consistently reported reductions in maternal hemoglobin and hematocrit after FLP with the most pronounced decrease typically observed within the first postoperative day [14]. For instance, Morikawa M’s study showed a decrease of hemoglobin from11.0 ± 1.0 to 9.3 ± 0.9 g/dL and hematocrit from 32.1 ± 3.0% to 27.3 ± 2.7% before and one day after FLP [15]. Similarly, Greimel P’s study documented a mean hemoglobin drop from 11.6 to 9.6 g/dl and a hematocrit decrease from 33.56% to 27.78% after FLP [8]. Our data align with these findings, showing a decrease in hemoglobin from 11.0 to 9.5 g/dL and in hematocrit from 33.4% to 28.3% one day after FLP in TTTS cases. We further identified a positive association between the volume of amniodrainage and the magnitude of hemoglobin and hematocrit decline, supporting the role of maternal intravascular dilution in these changes.

Notably, comparing with TTTS-cases without persistent compression, TTTS-cases with compression showed a prevention of hemoglobin (1.1 versus 1.5 g/dL, *p* = 0.014) and hematocrit drop (3.1% versus 4.0%, *p* = 0.05) after FLP. Additionally, this study showed a difference in the rate of blood transfusion in TTTS-cases with and without persistent compression (7.7% versus 2.2%) although it did not achieve significance which might be due to the limited sample size. Taken together, these findings suggest that persistent abdominal and uterine compression may help attenuate the hemodilution effect following FLP.

In addition to mitigating hemodilution, persistent compression at the trocar insertion site may also help prevent procedure-related bleeding. Although severe maternal complications are uncommon with FLP [16], the risk of uncontrollable bleeding from the uterine wall at the trocar insertion site warrants attention. Susumu Murata reported a case underwent caesarean hysterectomy after FLP because of massive bleeding from the uterine wall due to endoscopic insertion [12]. Consequently, the authors suggested to observe for about half an hour or one hour in the operation or recovery room after laser surgery. In our unit, a pregnant woman complicated with TTTS stage 2 developed hemorrhagic shock after FLP. Hemorrhage from an injury lesion where an endoscope had been inserted was confirmed during the emergency cesarean section. The patient was transferred to the intensive care unit and treated with a massive transfusion of 600 ml red blood cell concentrates. After this case, we implanted the practice of applying persistent gauze and bandage compression at the trocar insertion site to prevent uterine bleeding in our unit. There are some other methods reported previously to reduce the risk of hemorrhage related to the surgery. In some unit, operators routinely conduct transabdominal ultrasound examinations 30 min after FLP to see if there is a blood clot pooling in the mother’s intraperitoneal cavity to exclude the hemorrhage from injury of the uterine wall [13]. In some centers, collagen plug is placed at the insertion site to decrease such bleeding [7].

In this study, we mainly focused on the effect of compression on maternal blood dynamic changes while such maternal uterine compression could also play a role in decreasing the risk of amniotic fluid leakage from the amniotic cavity to the maternal peritoneal cavity, which need further evaluation. While compression demonstrated measurable effects on maternal hemoglobin and hematocrit changes, we did not observe corresponding improvements in maternal or fetal perinatal outcomes.

### Strengths and limitations

To our knowledge, it is the first study to investigate the impact of persistent compression on maternal hemodynamic changes following FLP. Our study showed a decrease in hemoglobin and hematocrit could be mitigated by persistent compression on the uterine insertion site after FLP. Even small reductions may be clinically meaningful for women at higher risk of anemia or limited transfusion resources. Moreover, the technique is simple, low-cost, and safe, supporting its feasibility as a preventive strategy during FLP. However, several limitations must be acknowledged. The study was retrospective and conducted across two centers, limiting the ability to obtain complete hematologic data for all patients. Factors such as surgical learning curve and variability in perioperative management may also have influenced the outcomes. Moreover, given the rarity of TTTS and the limited number of participating centers, the sample size, particularly for cases requiring transfusion was small, reducing the statistical power and generalizability of the findings. Further prospective studies with larger cohorts are needed.

## Conclusion

Although severe maternal complications following intrauterine interventions are relatively uncommon, surgeons should remain vigilant for postoperative bleeding and hemodynamic instability. Persistent compression at the uterine insertion site is a simple, low-cost, and practical technique that may mitigate postoperative declines in hemoglobin and hematocrit, thereby enhancing maternal safety following FLP.

## Supplementary Information

Supplementary Material 1.

## Data Availability

The data that support the findings of this study are available on request from the corresponding author. The data are not publicly available due to privacy restrictions.
