# Supplementary material for: Persistent compression of uterine insertion on maternal blood dynamic change in fetoscopic laser photocoagulation surgery: a novel method
Source: BMC Pregnancy Childbirth. 2026 May 13;26:730. doi: 10.1186/s12884-026-09227-6 (PMC13343597; doi:10.1186/s12884-026-09227-6)
Supplement: Supplementary file 1 — Supplementary Material 1. [file 12884_2026_9227_MOESM1_ESM.docx]

Supplementary Table 1. The values of hemoglobin and hematocrit before and after FLP in women with and without persistent compression in Drum Tower Hospital.

| Parameters | Persistent compression Group  N=46 | Non- persistent compression Group  N=11 | P value |
| --- | --- | --- | --- |
| Hemoglobin before FLP, g/dl, mean, SD | 10.7, 1.0 | 11.1, 1.2 | 0.281 |
| Hemoglobin after FLP, g/dl, mean, SD | 9.6, 1.1 | 9.3, 1.4 | 0.470 |
| Change of Hemoglobin, g/dl, mean, SD | 1.1, 0.8 | 1.8, 1.0 | 0.019 |
| Hematocrit before FLP, %, mean, SD | 32.3, 3.0 | 32.9, 3.2 | 0.560 |
| Hematocrit after FLP,  %, mean, SD | 29.3, 3.1 | 28.1, 3.4 | 0.274 |
| Change of Hematocrit,  %, mean, SD | 3.1, 2.5 | 4.9, 3.3 | 0.054 |
| Blood Transfusion after the procedure  n, % | 1/46, 2.2 | 1/11, 9.1 | 0.352 |

SD: standard deviation; FLP: Fetoscopic laser photocoagulation.

Supplementary Table 2. The values of hemoglobin and hematocrit before and after FLP in women without persistent compression in Drum Tower Hospital and Chongqing Hospital.

| Parameters | Non- persistent compression Group  In CQH  N=54 | Non- persistent compression Group  In DTH  N=11 | P value |
| --- | --- | --- | --- |
| Hemoglobin before FLP, g/dl, mean, SD | 10.4, 1.1 | 11.1, 1.2 | 0.098 |
| Hemoglobin after FLP, g/dl, mean, SD | 9.1, 1.1 | 9.3, 1.4 | 0.500 |
| Change of Hemoglobin, g/dl, mean, SD | 1.4, 0.6 | 1.8, 1.0 | 0.281 |
| Hematocrit before FLP, %, mean, SD | 31.0, 3.0 | 32.9, 3.2 | 0.067 |
| Hematocrit after FLP,  %, mean, SD | 27.1, 3.2 | 28.1, 3.4 | 0.384 |
| Change of Hematocrit,  %, mean, SD | 3.9, 2.3 | 4.9, 3.3 | 0.245 |
| Blood Transfusion after the procedure  n, % | 4/54, 7.4 | 1/11, 9.1 | 1.000 |
